# Bringing Self-Kindness Into the Workplace: Exploring the Mediating Role of Self-Compassion in the Associations Between Attachment and Organizational Outcomes

**DOI:** 10.3389/fpsyg.2019.01148

**Published:** 2019-05-21

**Authors:** Abira Reizer

**Affiliations:** Department of Psychology, Ariel University, Ariel, Israel

**Keywords:** avoidance attachment style, anxious attachment style, self-compassion, job performance, OCB, turnover intentions, emotional exhaustion, SEM

## Abstract

Research has shown that individual differences in adult attachment predict several organizational outcomes. However, little is known about the mechanism that underlies these associations. The current study examines whether self-compassion can serve as a potential mediator explaining the associations between individual differences in attachment and organizational outcomes. Four outcome measures were evaluated: job performance (HPQ; Kessler et al., 2003), organizational citizenship behaviors (OCB) (Goodman and Svyantek, 1999), turnover intentions (Abrams et al., 1998), and emotional exhaustion (Schaufeli et al., 1996). In addition, participants (*N* = 202, response rate 81%) also completed several questionnaires assessing attachment style (ECR; Brennan et al., 1998) and self-compassion (SCS; Neff, 2003). Using structural equation modeling (SEM) for testing the research hypotheses, the hypothesized model was supported, with self-compassion mediating the relationship between attachment styles and all four work-related outcomes. The research findings suggest that self-compassion can provide a solid mechanism for understanding organizational outcomes and for understanding individual differences related to attachment functioning in the workplace.

## Introduction

Attachment theory (Bowlby, 1982) provides a fundamental relational framework for understanding how people experience their close relationships, interpret the other’s intentions, and how they regulate their affect and behaviors in social settings (Collins et al., 2006; Mikulincer and Shaver, 2017; Fraley, 2019). In the last decade, a growing interest in attachment at the workplace has been seen as part of a general trend in the organizational domain to focus on cooperative relationships between the employees (Paetzold, 2015). This interest is part of an increased recognition that relationships comprise social capital and are fundamental for the success of the organizations (Yip et al., 2018). Indeed, recent accumulating evidence has indicated that individual differences in attachment predict employee functioning and organizational outcomes at the workplace, such as employment relationships (Albert et al., 2015; Yip et al., 2018), burnout (e.g., Reizer, 2015), organizational citizenship behaviors (OCB) (e.g., Harms et al., 2016), job performance (Ronen and Zuroff, 2017), and turnover intentions (e.g., Richards and Schat, 2011). This stream of research is still burgeoning, with nearly 50% of the papers on attachment at the workplace having been published after 2010 (Yip et al., 2018, p. 186).

Although prior research established the relationships between attachment styles and workplace outcomes, very little is known regarding the processes by which these associations occur (Harms, 2011; Harms et al., 2016). The current study goes beyond the direct associations between attachment and work-related outcomes by seeking to examine the potential mediating role of self-compassion, a relatively less explored mediator in the organizational field. *Self-compassion* is characterized as the capacity to express kind and compassionate feelings to oneself, including self-kindness, self-acceptance, mindfulness, and sense of common humanity upon facing difficulties or challenges (Neff, 2003). Specifically, the present study extends previous knowledge by introducing self-compassion, that may clarify the mechanism through which attachment affects various organizational outcomes.

### Attachment Dimensions as Predictors of Organizational Outcomes

Attachment theory is based on the notion that the ability to build close personal relationships may have a profound impact on one’s functioning in various interpersonal and social domains (Harms, 2011; Mikulincer and Shaver, 2017). In Bowlby’s (1982) terminology, attachment-relational dispositions represent the individual’s mental models of relationships with significant others. According to Bowlby (1982), early interactions between children and their significant caregiving figures provide the child with an inner sense of security. Children having experienced responsive and sensitive care, grow to feel safe and secure in the world and in their relationships with others. These internalized mental models comprise an inner knowledge that support will be available if needed and inner confidence they can handle life’s challenges. Therefore, this sense of security promotes social and personal adjustment (Bowlby, 1982; Mikulincer and Shaver, 2012).

However, insecurely attached individuals, who may have experienced intensive or inconsistent responsiveness from their significant caregivers (insecurely attached individuals) are prone to develop defensive perceptions of themselves and their interpersonal relationships (George and Solomon, 2008). They tend to express negative perceptions and behaviors regarding interpersonal relationships and are inclined to cope less effectively with stress and other life challenges (Lopez and Brennan, 2000; Mikulincer and Shaver, 2017).

Attachment theory was derived from examining parent-child relationships; however, it has been applied to enhance our understanding of adult relationships (Hazan and Shaver, 1987; Fraley, 2019; Fraley and Roisman, 2019). Researchers have conceptualized adult attachment insecurities in terms of a two-dimensional space: attachment anxiety and avoidance (e.g., Brennan et al., 1998). Anxiety develops when individuals experience ambivalent and inconsistent support. Over time, these experiences intensify feelings of helplessness and insecurity in evaluating others’ goodwill. Hence, anxiously attached individuals tend to express a lack of confidence regarding others’ reactions, perceive themselves as unworthy of love, and convey negative images of themselves in relationships. These individuals often become preoccupied with their relations, adopt behaviors aimed at eliciting affection and support from others (Mikulincer and Shaver, 2017; Fraley, 2019), and rely on others’ approval in order to maintain their self-esteem (Srivastava and Beer, 2005).

As for avoidance, these individuals have learned that other people cannot be trusted to be available to them in times of need or may reject their requests when approached. Thus, having received suboptimal care in their childhood leads them, as adults, to adopt a deactivating strategy of attachment needs. Therefore, they diminish their desire to receive support from others and instead rely heavily on themselves and on their personal resources (Ein-Dor et al., 2015; Mikulincer and Shaver, 2017). Individual differences in attachment styles have been shown to predict a wide variety of measures of relationship quality, mental health, stress management, and human functioning, as reported in hundreds of published studies (Mikulincer and Shaver, 2017, 2019; Fraley, 2019).

Attachment styles have particular importance in the workplace, as they represent the way employees perceive themselves and their colleagues in close and supportive interactions as well as their willingness to engage in such relationships (Fraley and Shaver, 2008). Hazan and Shaver (1990) expanded attachment theory to the domain of work relationships, suggesting that adult attachment manifestations at the workplace are similar to attachment dynamics in childhood. For example, they found that anxious employees tend to be preoccupied with relational and interpersonal issues and feel less appreciated. However, avoidant employees are likely to focus on non-relational aspects of work, as they are less satisfied with social interactions at the workplace and typically prefer to work alone. Organizational scholars have continued to advance research regarding attachment at the workplace. Of particular interest have been studies highlighting organizational outcomes, such as job performance, OCB, turnover intentions, and burnout (for a reviews, see Harms, 2011; Paetzold, 2015). In addition, OCB, job performance, employee retention, and employee burnout are considered as critical issues for organizations (for OCB, see Bolino et al., 2018, for performance, see Schleicher et al., 2018, 2019; for turnover, see Hancock et al., 2013; for burnout, see Montgomery et al., 2015). These outcomes become particularly important in the modern organization where the need to adapt and be competitive and dynamic are critical. In these organizations, success becomes even more dependent on the effective functioning of their employees.

#### Performance

While job performance may not immediately seem to be an outcome closely associated with attachment styles, it is one of the first studied aspects of organizational behavior in the attachment literature. As noted, Hazan and Shaver (1990) suggested that attachment insecurities are negatively associated with job performance. Anxiously attached individuals reported being unable to complete their job assignments, evaluated themselves lower on a self-ranking scale, and reported having the lowest average income. Avoidant individuals represent a different perspective at work. They tend to be less satisfied with their coworkers and evaluate themselves relatively low on job performance (Hazan and Shaver, 1990). Therefore, as both avoidant and anxious individuals presume that they cannot count on receiving the support they may need, they are less capable of meeting workplace challenges and demands and might be more distracted by conflicts and interpersonal matters (Mikulincer and Shaver, 2017). Surprisingly, empirical examinations of insecure attachment and job performance are scant. Additional support for these associations has shown that avoidance and anxiety were negatively associated with the amount of effort related to team tasks in a military setting (Rom and Mikulincer, 2003) as well as in an academic domain (Lavy et al., 2015). In addition, attachment security and job performance were shown to be mediated by workers’ trust in their supervisors (Simmons et al., 2009), coalition-building ability (Ronen and Zuroff, 2017).

Organizational citizenship behaviors (OCB) is defined as “the maintenance and enhancement of the social and psychological context that supports task performance” (Organ, 1997, p. 91). It has also been viewed as prosocial behavior at the workplace (Bolino and Grant, 2016). OCB represent both the extra-role behaviors directed toward the individuals within the organization as well as the behaviors directed toward helping the organization in general (Williams and Anderson, 1991). By acknowledging the interpersonal and discretionary aspects of OCB, previous studies have consistently supported the linkage between attachment and OCB (Syna Desivilya et al., 2006; Little et al., 2011; Paetzold, 2015). And indeed insecure employees they are less capable of building stable and trusting relationships, which impairs their willingness to make prosocial choices. It was indicated that anxious individuals tend to be preoccupied with their interpersonal relationships, finding it difficult to cope with workplace challenges that ultimately limit their ability and motivation to engage in discretionary behaviors such as OCB (Richards and Schat, 2011). As for avoidants, they tend to eschew social meetings and interactions, tend to be more suspicious of others’ intentions, and do not acknowledge the value of interacting or helping others, ultimately reducing the prospect of their OCB (Harms et al., 2016). Finally, it has been suggested that the association between anxiety and OCB is mediated by vigor (Little et al., 2011), while affective trust mediated the link between avoidance and interpersonal OCB (Harms et al., 2016).

#### Emotional Exhaustion

Burnout is considered a negative psychological outcome in the organizational domain (Alarcon, 2011). It is an affective reaction to ongoing stress, comprising emotional exhaustion, physical fatigue, and cognitive weariness (Shirom, 2003). Emotional exhaustion is recognized as a central component in work-related burnout measures (Qiao and Schaufeli, 2011). Robust effects have been established between employee insecurities (both avoidance and anxiety) and job burnout. Hazan and Shaver (1990) suggested that attachment styles would influence the emotional reactions to stressful workplace situations and to the ability to regulate work demands. And indeed, it has been shown that avoidance and anxiety are positively associated with burnout (Ronen and Mikulincer, 2009; Reizer, 2015) and with emotional exhaustion (Chopik, 2015). Specifically, anxious individuals feel overwhelmed by stressors at work, ultimately increasing their burnout and emotional exhaustion feelings. However, avoidant individuals tend to suppress their negative feelings at work and are not likely to approach others in times of need, thus eventually experiencing increased levels of emotional exhaustion as well (Chopik, 2015). Earlier efforts to investigate the mediation of the association between attachment and burnout through an organizational lens have shown that team cohesion (Ronen and Mikulincer, 2009) and social rejection (Ronen and Baldwin, 2010) mediated the links between anxiety and burnout. In addition perception of organizational fairness mediated the link between avoidance and burnout (Ronen and Mikulincer, 2009). However, the psychological mechanisms of these associations have yet to be examined.

#### Turnover Intentions

Relations with people at the workplace and the ability to socially interact with other employees are particularly relevant to the turnover literature. Research has consistently suggested that when people feel emotionally connected to others and develop social networks, they are less likely to express intentions of quitting the organization (Soltis et al., 2013) and are less likely to leave the organization (Hom et al., 2017; Rubenstein et al., 2018). As previously indicated, insecurely attached employees have considerable difficulty in forming affective emotional bonds with others in the organization. While anxious individuals are more involved with interpersonal conflict at work, avoidant individuals tend to distrust others, which diminishes their ability to form effective interpersonal relationships at the workplace (Hazan and Shaver, 1990; Harms, 2011). As social ties and work embeddedness provide some protection from turnover thoughts and actions (Lee et al., 2004), it is reasonable to assume that both anxiety and avoidance are negatively associated with turnover intentions. However, only scant published studies have sought to link attachment with turnover intentions, revealing inconsistent findings. Some studies have suggested that anxiety (and not avoidance) is a predictor of turnover intentions (Richards and Schat, 2011; Tziner et al., 2014). Conversely, Mikulincer and Shaver (2007) suggested that avoidance (and not anxiety) is associated with intentions to quit among high tech employees. In addition, other investigations have failed to find either anxiety or avoidance as associated with turnover intentions (e.g., Dahling and Librizzi, 2015) nor with any mediating processes (Tziner et al., 2014).

The current study explores the process through which attachment affects several workplace outcomes. In light of previous work, the first hypothesis focuses on the direct associations between attachment and workplace outcomes. In addition, by expanding the accumulating knowledge of the mediating mechanisms of attachment at the workplace (e.g., Ronen and Mikulincer, 2009; Simmons et al., 2009; Harms et al., 2016; Ronen and Zuroff, 2017), the current study explores the mediating effects of self compassion.

### Self-Compassion as a Predictor of Organizational Outcomes

Self-compassion represents a compassionate, kind, and caring attitude toward the self when facing difficulties, painful circumstances, or personal failures (Neff, 2003). Self-compassion comprises three primary components: (1) *Self-kindness* represents gentle, caring, and non-judgmental acceptance of oneself; (2) *A sense of common humanity* is the capacity to recognize the shared human experience. It includes framing out imperfect experiences and accepting that all people may struggle in some way or another, may fail, and may have to face problems and difficulties; (3) *Mindfulness* represents a clear and balanced approach to the immediate moments without suppressing or ruminating on them. Furthermore, three uncompassionate dimensions are expected to be less prominent among self-compassionate individuals: (1) *self-judgment* (harsh blame and judgment toward the self for not being good enough or for unsuccessfully coping with life difficulties); (2) *isolation* (focusing on the individual’s feeling of isolation from others rather than feelings of connection); and (3) *overidentification* (expressing an unbalanced approach regarding one’s difficulties, such as being caught up and swept away by negative experiences, feelings, and thoughts; Neff, 2003; Neff et al., 2017). These six elements can be merged into a single general score of self-compassion, by reverse-scoring the negative dimensions (Neff et al., 2017).

Over the past decade, clinical and social research has considered self-compassion as a positive psychological strength and a source of happiness. Recent meta-analyses have provided consistent and significant empirical support indicating that self-compassion is positively associated with personal well-being (for a meta-analysis, see Zessin et al., 2015) and negatively associated with psychopathology (MacBeth and Gumley, 2012). For example, self-compassion has been shown to decrease anxiety and depression, even when controlling for negative affect and self-criticism (Neff, 2003; Neff and Germer, 2017).

However, despite the significant impact of self-compassion in clinical and social settings, the role of self-compassion in the organizational domain has been seldom examined, thus remaining a promising area of research in the organizational sciences (Dev et al., 2018). Specifically, with self-compassion being viewed as a resilience factor and as a valid predictor of well-being, empirical evidence has revealed associations between self-compassion, emotional exhaustion, and burnout at the workplace (Alkema et al., 2008; Raab, 2014; Dev et al., 2018). Furthermore, self-compassion enhances performance as it offers a pathway to overcome mental barriers, aversive thoughts, fear of failure, and negative emotions (Neff and Knox, 2017). Consequently, self-compassion facilitates sport performance goals (e.g., Killham et al., 2018), academic demands (Leary et al., 2007), and performing laboratory tasks (Breines and Chen, 2012).

Self-compassion may also promote prosocial behaviors at work. Compared with those lower in self-compassion, high self-compassionate individuals are more emotional, connected, accepting, and less controlling partners (Neff and Beretvas, 2013). They tend to be more empathic and compassionate toward other people (Longe et al., 2010), and express more prosocial behaviors (Lindsay and Creswell, 2014). These qualities may contribute to their willingness to express more prosocial behaviors at work as well (such as OCB). Self-compassion can also be associated with persistence and lower levels of turnover intentions. It was indicated by an experimental investigation showing the effect of self-compassion on increased persistence and self-improvement motivation following initial failure in the laboratory (Breines and Chen, 2012). However, more research is needed to solidify the evidence for the manifestation of self-compassion at the workplace.

### The Meditating Role of Self-Compassion

The current study contributes to the existing literature by exploring a mechanism derived from the clinical and social literature as constituting the process through which attachment impacts organizational outcomes. The theoretical construct of self-compassion has been described as stemming from individual differences in attachment, being that early family and parenting experiences (Wei et al., 2011; Germer and Neff, 2015). Supportive parenting serves as a caring and comforting model fostering self-compassion. However, critical parenthood, dysfunctional family life, or early dysfunctional attachment experiences do not provide any positive and compassionate models upon which to draw, thus impairing self-compassionate perceptions (Gilbert, 2009; Germer and Neff, 2015). Indeed, both anxiety and avoidance-insecure attachment dimensions are negatively associated with the supportive self-accepting approach of the self-compassion mindset (Neff and McGeehee, 2010; Wei et al., 2011). Furthermore, previous work has indicated that avoidance and anxiety are predictive of subjective well-being (Wei et al., 2011) and mental health (Raque-Bogdan et al., 2011) through the mediating role of self-compassion.

By expanding the knowledge of the mechanisms of attachment, the current study suggests that self-compassion can explain the effect of attachment on diverse organizational outcomes. Anxiously attached employees tend to develop a negative view of themselves at the workplace and report feelings of being unevaluated there (Hazan and Shaver, 1990). They tend to report increased negative feelings at work (Krpalek et al., 2014), accompanied by a preoccupation with these negative thoughts and feelings (Kafetsios et al., 2014). In addition, anxious individuals perceive negative emotions and events in an idiosyncratic manner as something that happens only to them (Wei et al., 2011). These tendencies eventually impair their capacity to experience self-compassion, which, in turn, decrease their functioning at the workplace.

With respect to avoidant individuals, they appear to distract themselves from their inner emotional world at their workplace by engaging in surface acting or suppression (Richards and Schat, 2011; Kafetsios et al., 2014). They also prefer to detach themselves instead of attaining some comfort and support from others (Harms, 2011; Richards and Schat, 2011). These qualities impair their ability to manage their own emotions more effectively and decrease their self-compassionate and caring perceptions toward themselves. This tendency may manifest itself in lower organizational outcomes.

### Research Objectives and Hypothesis Forming

Accumulating evidence in the organizational literature has highlighted the role of attachment personality styles in predicting several organizational outcomes (such as performance, OCB, emotional exhaustion, and turnover intentions). Although prior research established the relationships between attachment styles and workplace outcomes, very little is known regarding the processes by which these associations occur. Integrative reviews have advocated that the next step in the study of attachment at the workplace should focus on the potential mediators of attachment styles in the organization (Harms, 2011; Harms et al., 2016; Yip et al., 2018). Thus, the current study set two primary goals. First, I sought to examine the role of attachment avoidance and anxiety in predicting diverse organizational outcomes. While some organizational outcomes, such as OCB and emotional exhaustion, have received robust support in the literature (e.g., Little et al., 2011; Chopik, 2015), others, such as turnover intentions, have produced less conclusive findings (Tziner et al., 2014) and still require further empirical support. Aiming to expand previous work, the first hypothesis examines whether attachment avoidance and anxiety will decrease OCB and job performance measures and increase emotional exhaustion and turnover intentions.

H1: *Attachment anxiety and attachment avoidance will decrease OCB and job performance and will increase turnover intentions and emotional exhaustion.*

Second, valuable insights into the mediating mechanism that manifests attachment in the organizational domain are still lacking (Harms, 2011; Ronen and Zuroff, 2017). Previous personality-centered research among students and the general population has suggested that self-compassion serves as a potential mediator of attachment processes (e.g., Wei et al., 2011). Thus, based on the theoretical background, the second hypothesis examines the mediating role of self-compassion in the associations between attachment and organizational outcomes (OCB, performance, emotional exhaustion, and turnover intentions).

H2: Self-compassion mediates the associations between attachment dimensions and the organizational outcomes of job performance, OCB, turnover intentions, and emotional exhaustion.

## Materials and Methods

### Participants and Procedure

Data were collected from 202 Israeli service-sector employees who had been employed for at least 2 months. The mean organizational tenure was 3.69 years (*SD*_tenure_ = 2.56). Among the participants, 73% were women, and 49% had an academic degree (the remainder were high school graduates). The employees weekly working hours averaged 30.88 (*SD*_workhours_ = 12.90). The participants were relatively young (*M*_age_ = 27.93, *SD*_age_ = 9.12), as Israeli young adults are primarily employed in service jobs (Fuchs, 2015). The study was approved by the Ethics committee of the Social Sciences and Humanities at Ariel University. Data were collected during March and April 2014. Three trained research assistants recruited the participants to take part in an online survey in two ways. First, personal contacts were approached to identify participants with a request to take part in the study. In addition, announcements were dispersed in campus social media outlets. The online link insured anonymity, as neither the researchers nor the research assistants had access to the participants’ personal data. Participation in the study was entirely voluntary. Prior to administering the online measures, an email was sent to all participants, explaining the aims of the research, the voluntary nature of participation, potential benefits and risks, and data confidentiality. Participants were informed of their right to withdraw from the study at any stage. After reading an informed consent page, the participants were requested to approve it by clicking the acceptance box at the bottom of the agreement.

Participation in the online survey was activated by means of an unidentifiable link. The time needed to complete the questionnaires was approximately 30 min. Online survey links were sent to 250 participants, with 214 returning completed forms. After an initial screening for missing data, eight participants with missing responses higher than 30% were eliminated from the data set, given that severe rates of missing data might bias the results (Schlomer et al., 2010). An additional four participants were also excluded from the analysis due to their not matching the inclusion criterion of working in the service sector, resulting in a final sample of 202 employees (response rate of 81%). No significant differences were found between the excluded and the final groups in age, *t*(210) = -0.30, *p* = 0.77, gender χ^2^(1) = 0.31, *p* = 0.73, and in job tenure, *t*(75) = 1.25, *p* = 0.21. However, a significant difference was revealed in number of weekly work hours, *t*(208) = 2.26, *p* = 0.03, indicating that the exclusion group members work fewer hours per week (*M*_workhours_ = 16.50) than do the final sample group (*M*_workhours_ = 30.88), thus, providing further justification for their exclusion.

#### Attachment Insecurity

Attachment anxiety and avoidance were assessed with the Experiences in Close Relationships scales (ECR; Brennan et al., 1998). Participants rated the extent to which each item was descriptive of their experiences in close relationships, presented on a 7-point Likert-type scale, ranging from 1 (*not at all*) to 7 (*very much*). For the current study, attachment dimensions were assessed with a short version (16-item) of the ECR (Ronen and Mikulincer, 2012). Eight items tapped attachment anxiety (e.g., “I worry about being abandoned”), and eight items tapped avoidance (e.g., “I prefer not to show a partner how I feel deep down”). Cronbach’s alphas of the ERC measurement reported by Ronen and Mikulincer (2012) were relatively high for anxiety and avoidance items, ranging from 0.84 to 0.85. Cronbach’s α for the current sample was 0.85 for the attachment anxiety and 0.81 for the avoidance.

#### Self-Compassion

The Self-Compassion Scale (SCS; Neff, 2003) is a 26-item questionnaire. Participants rated the extent to which they behave in the manner indicated by each of the items (e.g., “I try to be loving toward myself when I’m feeling emotional pain”). The SCS comprises six subscales: Self-Kindness (five items), Self-Judgment (five items), Common Humanity (four items), Isolation (four items), Mindfulness (four items), and Over-Identified (four items). The items are rated on a 5-point Likert-type scale, ranging from 1 (*almost never*) to 5 (*almost always*). This scale has been widely used in different contexts and in various languages (Neff and Knox, 2017). A total SCS score has been recommended for use as a global measure of self-compassion (Wei et al., 2011; Neff et al., 2017). Thus, for the current study, the total SCS score was adopted as the self-compassion measure for the analysis. Higher scores indicate a higher level of self-compassion. Neff et al. (2003) provided extended support for the validity and reliability of the scale (internal consistency for the original SCS scale was 0.92). The scale’s Cronbach α for the current sample was 0.89.

#### Turnover Intentions

We adapted Abrams et al.’s (1998) three item scale to assess turnover intentions (“In the next few years, I intend to leave the company”; “In the next few years, I expect to leave the company”; “I think about leaving this company”). Questionnaire items were presented on a 5-point Likert-type scale, ranging from 1 (*strongly disagree*) to 5 (*strongly agree*). Higher scores reflect greater intentions to leave the organization. Cronbach’s α in the original scale was 0.88 and was even higher for the present sample, at 0.94.

#### Job Performance

Job performance was assessed with three items derived from the Health and Work-Performance Questionnaire (HPQ; Kessler et al., 2003, 2004). The HPQ is a reliable and valid self-rated work-performance measure, scored as percentage of performance on a 0–10 response scale (e.g., “Using the same 0–10 scale, how would you rate your overall job performance on the days you worked during the past 4 weeks?”). Performance score was obtained by multiplying the respondent’s response by ten, as recommended by Kessler et al. (2003). The original Cronbach’s alpha for this scale was 0.74 for the reservation agents and 0.81 for the customer service representatives assessed by Kessler et al. (2003). Cronbach’s α for the current service sector sample was 0.81.

#### Organizational Citizenship Behaviors (OCB)

Organizational citizenship behaviors was measured using eight items from Goodman and Svyantek’s scale (1999). Participants were asked to indicate the extent to which they found these statements characteristic of themselves on a 6-point Likert-type scale, ranging from 1 (*not at all characteristic*) to 6 (*totally characteristic*). Sample items include, “Willingly attend functions that are not part of the job, helps in the overall image of the organization”; “Help others when their workload increases (assists others until they get over the hurdles)”; “Help colleagues with their work when they have been absent.” Higher scores reflect greater OCB. Goodman and Svyantek (1999) provided solid support for the validity and the reliability of the scale. Cronbach alpha for the total OCB was 0.89 for their sample. Cronbach’s α for the OCB measure for the current sample was 0.87.

#### Emotional Exhaustion

Emotional exhaustion was measured by five emotional exhaustion items from the Maslach Burnout Inventory, as adapted for the occupational context (MBI-GS, Schaufeli et al., 1996; e.g., “I feel emotionally drained from my work”). All items are scored on a 7-point Likert-type scale, ranging from 0 (*never*) to 6 (*always*). Schaufeli et al. (1996) reported satisfactory internal consistency of the scale, with the original Cronbach alpha coefficients ranging from 0.84 to 0.90. Cronbach’s α for the current sample was 0.81.

### Control Variables

Previous literature underscored the fact that gender may be differentially impacted by attachment and self-compassion (Kirkpatrick and Davis, 1994; Yarnell et al., 2015). Specifically, self-compassion appears to increase with age (Bratt and Fagerström, 2019), and age is negatively correlated with attachment anxiety (Chopik et al., 2013). Therefore, to ensure a fuller understanding of how attachment style differences influence organizational outcomes, gender and age require control.

## Results

### Preliminary Analysis and Descriptive Statistics

A confirmatory factor analysis (CFA) using SEM was performed prior to testing for the hypothesized model. The CFA assessed whether each of the measurement items loaded significantly onto the scales with which they were associated. The model comprised all the observed items loading on their respective seven latent factors: two independent variables (avoidance and anxiety), one mediator (self-compassion), and four dependent variables (turnover intentions, performance, OCB, and emotional exhaustion). A parceling procedure was used for the self-compassion measure. Self-compassion’s 26 items were assigned to six dimensions (Neff et al., 2017). Parceling serves to create several measures for the latent construct, thereby reducing the measurement error, reducing the risk of spurious correlations, and increasing scale points (Little et al., 2002). Items were assigned to the percentiles using Neff et al.’s (2017) recommendation. The parcels were found to be normally distributed. The measurement model showed an acceptable fit with the data, χ^2^(751) = 1201.66, *p* = 0.00, χ^2^/*df* = 1.60, CFI = 0.91, NFI = 0.91, TLI = 0.90, and RMSEA = 0.055. Overall, the CFA fit indices support the hypothesized model (Hair et al., 2009; Jackson et al., 2009).

The means, standard deviations, and correlations between research variables are presented in Table 1. Attachment anxiety and attachment avoidance were significantly associated with OCB and emotional exhaustion. In addition, anxiety was positively associated with turnover intentions, and avoidance was negatively associated with job performance. These significant correlations support H1. The associations between attachment styles (both anxious and avoidance) and self-compassion were negative and significant. Finally, self-compassion was negatively associated with emotional exhaustion and turnover, but positively associated with job performance and OCB. Thus, the correlations confirmed the research hypotheses. Finally, the correlations between anxiety and job performance, as well as between avoidance and turnover intentions, did not achieve significance. However, it has been strongly suggested that researchers may proceed with mediation analysis, even when the direct relationships are non-significant, as indirect effects can provide different outcomes (Shrout and Bolger, 2002; Little et al., 2011). Age was negatively associated with anxiety and turnover intentions and positively associated with OCB.

**Table 1:** Means, standard deviations, and correlations.

	Mean	*SD*	1	2	3	4	5	6	7	8	9
(1) Avoidance	2.80	1.03	(0.81)								
(2) Anxiety	3.29	1.26	0.41***	(0.85)							
(3) Self-compassion	3.14	0.58	-0.36***	-0.42***	(0.89)						
(4) Job performance	73.05	16.58	-0.17*	-0.06	0.21**	(0.81)					
(5) OCB	4.45	1.01	-0.23**	-0.12*	0.31***	0.46***	(0.89)				
(6) Turnover intentions	3.50	1.37	0.07	0.18**	-0.21**	-0.09	-0.31***	(0.94)			
(7) Emotional exhaustion	2.43	1.59	-0.19**	0.22**	-0.30***	-0.22**	-0.43***	0.60***	(0.81)		
(8) Age	27.93	9.12	0.01	-0.19**	0.11	0.01	0.16*	-0.30***	0.13	–	
(10) Tenure	3.69	2.56	0.05	-0.18*	0.12	0.07	0.16*	-0.15*	-0.06	0.62***	–
(11) Gender	–	–	-0.12	0.07	0.03	0.02	0.12	0.09	-0.03	-0.29***	-0.23**


**^∗^p < 0.05, ^∗∗^p < 0.01, ^∗∗∗^p < 0.001. Values in parentheses are reliabilities.**

### Model Testing

Structural equation modeling (SEM; Arbuckle, 2010) was used to examine the current research hypotheses while controlling for age and gender. In order to assess the model fit, the following indices were used: χ^2^, χ^2^/df index, normed fit index (NFI), comparative fit index (CFI), Tucker–Lewis index (TLI), and root-mean-square error of approximation (RMSEA). Model fit with NFI, CFI, and TLI was equal or greater than 0.90, RMSEA equal to or less than 0.08, and χ^2^/df index < 3 are indicative of an adequate fit to the data (Hair et al., 2009; Awang, 2012).

Three structural equation models were examined to determine which of the models provided the best fit to data. The first model included indirect paths between attachment and four organizational outcomes (performance, OCB, turnover intentions, and emotional exhaustion), mediated by self-compassion. The second model included direct and indirect paths between attachment styles and the same outcomes, through self-compassion, in addition to a direct path between attachment and the organizational outcomes. The third model examined the direct paths between attachment styles and workplace outcomes. Although the alternative models indicated good fit indices (see Table 2), the results of the hypothesized model provided the best results, χ^2^(16) = 25.55; χ^2^/*df* = 1.60, *p* = 0.06; TLI = 0.934; CFI = 0.971; NFI = 0.931; RMSEA = 0.055, indicating good fit. A comparison of the hypothesized model with the alternative direct and indirect models indicated that the alternative model did not improve fit, Δχ^2^(6) = 6.84, *p* > 0.05, nor did the second alternative model, Δχ^2^(6) = 4.34, *p* > 0.05. Moreover, all the direct associations between attachment and organizational outcomes were non-significant. Specifically, non-significant associations were revealed between avoidance and job performance (β = -0.14, *p* = 0.08), OCB (β = -0.06, *p* = 0.33), turnover intentions (β = -0.01, *p* = 0.94), and emotional exhaustion (β = 0.08 *p* = 0.27).

**Table 2:** Comparison of alternative path models.

Model test	χ^2^	*P*	*df*	χ^2^/*df*	CFI	NFI	TLI	RMSEA
(1) Hypothesized Model: Indirect path from attachment to outcomes mediated by self-compassion	14.10	0.06	10	1.41	0.98	0.96	0.96	0.05
(2) Alternative Model 1: indirect path from attachment to outcomes and direct path to outcomes	7.26	0.12	4	1.82	0.99	0.97	0.91	0.06
(3) Alternative Model 2: direct paths from attachment to outcomes	9.76	0.04	4	2.44	0.97	0.96	0.85	0.09


**Age and gender are controlled for all three models*.*

**FIGURE 1:**
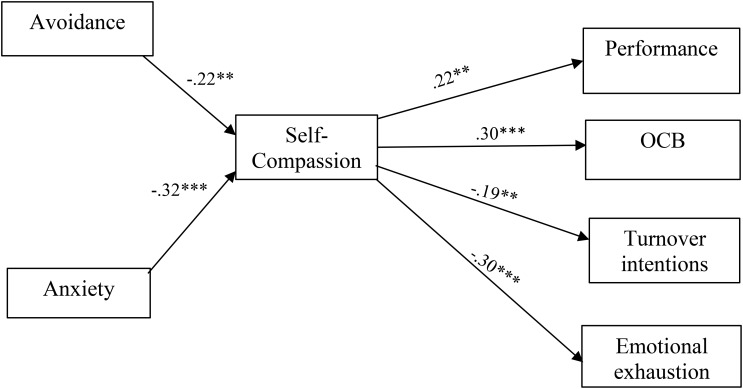
Mediation effects of self-compassion in the associations between attachment and organizational outcomes. ^∗∗^*p* < 0.01, ^∗∗∗^*p* < 0.001.

Similarly, non-significant associations were revealed between attachment anxiety and job performance (β = 0.08, *p* = 0.35), OCB (β = 0.08, *p* = 0.33), turnover intentions (β = 0.07, *p* = 0.36) and emotional exhaustion (β = 0.07, *p* = 0.39). This implies that the direct associations between attachment and organizational outcomes are fully mediated by self-compassion. Therefore, the full mediation model is the preferred one, as it presents a more parsimonious result. This final model is presented in Figure 1.

In order to examine the mediation hypotheses, the bootstrap technique using the confidence-interval method was applied. This technique is highly recommended, as it does not rely on distribution assumptions and can be applied when the assumptions of large sample size and multivariate normality may not hold (Ryu and Cheong, 2017). Bias correction bootstrapping is a resampling method that involves creating a sampling distribution to estimate standard errors and to create the confidence intervals. Bootstrapping confirms the mediation effect by assessing the confidence intervals for the indirect effect. If the confidence intervals do not include zero, then the null hypothesis is rejected, and the mediation effect is non-zero. Previous work referred to it as the preferred and relatively powerful approach for assessing mediation effects (Ryu and Cheong, 2017).

Supporting H2, the indirect effects of attachment on organizational outcomes through self-compassion were significant. Self-compassion mediated the effect of avoidance on job performance (*indirect effect* = -0.05, *p* = 0.003, *95% CI* = [-0.10, -01], *p* = 0.003), OCB (*indirect effect* = -0.07, *p* = 0.003, *95% CI* = [-0.12, -0.02]), turnover intentions (*indirect effect* = 0.04, *p* = 0.004, *95% CI* = [0.02, 0.09] ), and emotional exhaustion (*indirect effect* = 0.07, *p* = 0.003, *95% CI* = [0.01, 0.12]). In addition, self-compassion mediated the effect of anxiety on job performance (*indirect effect* = -07, *p* = 0.001, *95% CI* = [-0.012, -0.03]), OCB (*indirect effect* = -0.09, *p* = 0.0001, *95% CI* = [-0.16, -0.05]), turnover intentions (*indirect effect* = 0.06, *p* = 0.003 *95% CI* = [0.2, 0.111]), and emotional exhaustion (*indirect effect* = 0.10, *p* = 0.0001, *95% CI* = [0.05, 0.16]). The findings of the hypothesized model support the mediating role of self-compassion (see Table 3) in the relationship between attachment styles (avoidance and anxiety) and job performance, OCB, turnover intentions, and emotional exhaustion.

**Table 3:** Indirect effects and 95% Confidence Interval in the Hypothesized Mediation Model.

			95% BC	95% BC
Indirect path	Effect	*SE*	confidence LL	confidence UL
Avoidance → Self-compassion → Performance	-0.05**	0.02	-0.10	-0.01
Avoidance → Self-compassion → OCB	-0.07**	0.03	-0.12	-0.02
Avoidance → Self-compassion → Turnover Intentions	0.04**	0.02	0.02	0.09
Avoidance → Self-compassion → Emotional exhaustion	0.07**	0.03	0.01	0.12
Anxiety → Self-compassion → Performance	-0.07**	0.02	-0.12	-0.03
Anxiety → Self-compassion → OCB	-0.09***	0.03	-0.16	-0.05
Anxiety → Self-compassion → Turnover Intentions	0.06**	0.02	0.02	0.11
Anxiety → Self-compassion → Emotional exhaustion	0.10***	0.02	0.05	0.16


*^∗∗^p < 0.01, ^∗∗∗^p < 0.001. Bootstrapping sample size = 5000. Age and gender are controlled in all models. SE, standard error; LL, bootstrapping lower limit confidence interval; UL, bootstrapping upper limit confidence interval.*

## Discussion

The present study contributes to the current literature in several ways. First, the findings contribute to the growing and expanding literature on attachment at the workplace (Yip et al., 2018). The results illustrate the indirect effects of self-compassion as a dominant process regulating the negative effects of attachment insecurities on several organizational outcomes. Second, self-compassion was shown to facilitate positive aspects at work, such as job performance and OCB, and to decrease negative work experiences, such as emotional exhaustion and turnover intentions. Thus, the current findings extend previous social and clinical research and advance empirical understanding of the role of self-compassion at the workplace.

### The Mediating Role of Self-Compassion

The current findings shed new light by confirming the indirect mechanism of self-compassion linking individual attachment dimensions to several organizational outcomes. Only limited empirical work has been carried out regarding the associations between attachment dimensions and affect regulation at work, focusing mainly on the non-adaptive regulatory strategies used by avoidance and anxious individuals in managing their negative emotions. For example, avoidance and anxiety have been found to be associated with negative emotions at work (Kafetsios et al., 2014; Krpalek et al., 2014), and while avoidant individuals tend to use emotion suppression or surface-acting strategies (Richards and Schat, 2011), emotion rumination and reappraisal strategies are more common among the anxious (Mikulincer and Shaver, 2017).

Self-compassion differs from emotion regulation or emotion suppression mechanisms. Self-compassion does not avoid pain or seek to diminish, ignore, or amplify it, but rather enables the person to wrap one’s pain and difficulties in a warmer and accepting embrace and to generate positive feelings that help offset the negative ones (Neff, 2003; Neff and Knox, 2017). Therefore self-compassion might enhance psychological strength (Germer and Neff, 2015). For example, a person may still feel negative emotions such as fear, sadness, or anger, but can incorporate feelings of acceptance, love, or happiness that counterbalance the negative aspects. Alongside self-compassion’s role as a protective mechanism from feelings of guilt and shame outside workplace (Leary et al., 2007; Johnson and O’Brien, 2013), it may also function as a significant emotionally uplifting mechanism at the workplace.

The study’s findings can provide the organizational scholar with a better understanding of how self-compassion can matter at the workplace. The positive associations between self-compassion job performance and OCB and the negative associations with emotional exhaustion and turnover intention provide additional support to the cumulative research relating to self-compassion as a healthy and adaptive intrapersonal mechanism (Breines and Chen, 2012; Zhang and Chen, 2016). As the modern workplace provides the employee with endless challenges, barriers, cognitive and emotional demands, and other difficulties (Giorgi et al., 2017; Achnak et al., 2018), the ability to provide comforting self-love and compassion to oneself may ultimately expand one’s resources to face these workplace difficulties more effectively.

However, as only few studies have examined the role of self-compassion as a potential predictor in the workplace setting (e.g., Kreemers et al., 2018), the mechanism through which self-compassion affects job-related outcomes is as yet unclear. For instance, does self-compassion comprise an effective mechanism to cope with stress and an emotion facilitator, enabling the individual to embrace aversive events by incorporating the positive ones (Neff et al., 2005; Zhang and Chen, 2016) and recognizing mistakes without been overwhelmed by negative affect (Leary et al., 2007)? Alternatively, does self-compassion function as a motivational tool facilitating self-improvement efforts, as suggested by Breines and Chen (2012)? Indeed, self-compassion may serve as an enhancer to both aspects, with future studies likely to expand our understanding of the mechanisms linking self-compassion to organizational outcomes.

In addition, while previous theories have addressed the role of emotion regulation, such as emotional labor (Hochschild, 1983), in improving employee functioning, self-compassion offers a unique perspective in this area. Contrary to previous work that often stressed the notion that strong and effective employees should be stoic and silent regarding their own suffering and be able to suppress their feelings, self-compassion highlights the workers’ capacity to comfort themselves when they are hurting and are in need of care and affection (Neff and Knox, 2017). Thus, self-compassion can comprise an inner resource that can facilitate employees’ achieving hope and inner strength when confronting workplace challenges. Future research should investigate these areas of inquiry in addition to the underlying mechanisms associating self-compassion and workplace outcomes.

### Practical and Clinical Implications

Several potential practical implications arise from this research. First, the study may help raise awareness among organizational professionals regarding the ways in which insecure is associated with several work outcomes. Importantly, it is encouraging to note that managers, as well as work colleagues have been shown to comprise a secure base of assurance and trust and thus fulfill attachment needs for acceptance and closeness for the insecurely attached (Rom and Mikulincer, 2003; Mayseless and Popper, 2007) and ultimately enhancing the employee’s well-being and functioning. For example, it has been suggested that supervisors’ secure-based support may avert unintended negative consequences and increase proactivity, self-efficacy, and motivation among the insecurely attached employees (Wu and Parker, 2017). In addition, group and team cohesion and support may also increase performance among insecurely attached individuals (Rom and Mikulincer, 2003; Lavy et al., 2015). Thus, perhaps a horizontal transfer within the organization to a more cohesive team could suffice as an intervention to avert dysfunctional attachment relationships manifesting themselves into negative workplace behaviors. Finally, Hardy and Barkham (1994) found that avoidant and anxious employees improved their insecure attachment scores in the course of attachment-focused therapy. These results point to the potential benefits of security-building interventions in the workplace that may prove to be effective for those with insecure attachment styles and may decrease the negative effects of insecurity regarding workplace outcomes, found in the current work.

Secondly, the mediating role of self-compassion highlights the possibility that practical and clinical interventions need to be provided to insecurely attached individuals. These intervention prospects are promising, given that self-compassion has been shown to be trainable and, therefore, remediable (Kirby et al., 2017). Given the trainability of self-compassion (Shapira and Mongrain, 2010; Neff and Germer, 2013), employers and managers can foster more self-compassion by telling and reminding workers to be self-compassioned (Breines and Chen, 2012), by using meditation (Rao and Kemper, 2017) and mindfulness practices (Sirgy and Jackson, 2015) that enhance self-compassion (Zenner et al., 2014), or even by engaging in self-compassioned writing exercises (e.g., see Leary et al., 2007; Shapira and Mongrain, 2010; Zhang and Chen, 2016).

It seems that effective interventions, whether implemented by consultants or by organizational HR practitioners, should incorporate a module of self-compassion training. This training could improve the insecurely attached individual’s capacity to handle workplace challenges, thus leading to more effective functioning at work. In addition, anxious or avoidant individuals are seriously challenged in identifying, describing, and self-regulating their emotions (Barbasio and Granieri, 2013; Pepping et al., 2013; Mikulincer and Shaver, 2019). Therefore, it can be suggested that future interventions and clinical practices with insecurely attached individuals could address self-compassionate techniques that focus on the emotional aspects of the workplace. Moreover, future studies should seek to determine organizational and clinical interventions that can effectively decrease anxious distress feelings and thoughts and can alleviate avoidant distancing from emotional experiences and close relationships.

### Future Directions

The current study provides several promising directions for further research. An initial line of inquiry can be focused on the need to deepen the current understanding of the variables moderating and mediating the role of attachment at the workplace, which is recognized as one of the more prominent potential research domains of applied individual differences (Harms, 2011). Specifically, the current study demonstrates how anxious and avoidant employees relate to several workplace outcomes and offers a possible mechanism for explaining it. However, reaching a full understanding of the relations between attachment dimensions and organizational outcomes still comprises an important challenge for future investigators. For example, as recent work in attachment at the workplace has suggested that the effect of homogeneity or heterogeneity in attachment measures among team members can be critical for team performance (Lavy et al., 2015), it may be beneficial to incorporate the mediating role of self-compassion as a group construct in understanding attachment team dynamics. In addition, more mediating processes need to be investigated. As anxiety and avoidance are negatively associated with a sense of meaning in life (Reizer et al., 2013), one may investigate the contribution of sense of meaning, either in life or at work, in the associations between attachment and individual functioning at the workplace. Finally, future work might expand the current model and examine the mediating role of self-compassion in the associations between attachment and several other potential outcomes such and job satisfaction (Reizer, 2015) and job commitment (Yip et al., 2018).

A second line of inquiry would be to address expanding the role of self-compassion at the workplace. A common belief is that workers need to apply a harshly self-critical perspective in order to succeed in the modern workplace, yet the current study supports an opposite conclusion. In line with several findings derived from the academic context, it appears that self-compassion comprises several beneficial features. For example, in the academic setting, self-compassion has been positively associated with performance goals, diminished fear of failure, and a tendency to resilience when confronting failure (Neff and Knox, 2017). While self-compassion is negatively associated with perfectionism, it has no associations with the level of performance standards adopted by the self (Neff and Knox, 2017); this indicates that self-compassion does not promote passivity, but is consistent with striving to do one’s best by trying to avoid past mistakes (Breines and Chen, 2012). Though these promising findings are consistent with the demonstrated positive effects of self-compassion, the self-compassion construct and its components (such as mindfulness) are still considered only a niche in the organizational arena (Sutcliffe et al., 2016). Thus, there remains a need to further delineate the moderating and mediating mechanism of self-compassion at the workplace, address interesting theoretical questions, and identify the boundaries of the self-compassion effect. For instance, does self-compassion function uniformly in all occupations, and at all career stages? Can it be harmful or ineffective in certain circumstances, such as in unrealistically motivating employees in certain circumstances?

### Limitations

Placing the findings in perspective, some limitations of the current research need to be considered. First, the study is correlational in nature and data were derived from self-reports. Thus, causal conclusions should be drawn with caution. There is a need to be cognizant of possible influences of common method bias (Podsakoff et al., 2012). Nonetheless, future research could incorporate additional data sources (e.g., supervisors’ reports of actual OCB and job performance) to further offset confounding influences. A neuropsychological study using fMRI found that self-compassion is associated with neuronal activity similar to what transpires when feeling compassion and empathy toward others (Longe et al., 2010). This promising line of research suggests that neural activity can also be relevant in considering workplace outcomes, such as OCB or even burnout. In addition, although the study’s general population sample may bear some advantages, future research should examine whether the current findings hold for different levels of tenure, different organizations, and across different job and career stages. For example, self-compassion has been suggested as a trait that may facilitate better coping with the challenges of the job search process (Kreemers et al., 2018). Therefore, future work may focus on the role of self-compassion in the interactions between attachment and contextual stressors in coping with workplace challenges. Despite these limitations, the current findings frame a rich playing field for further exploring the mediating mechanisms of attachment at the workplace.

## Conclusion

Attachment theory is currently recognized as one of the most influential theories in developmental, personality, and social psychology. Nonetheless, it is an emerging area of interest among organizational scholars, and there is an increased need to understand the mediating processes of attachment at the workplace (Harms, 2011; Yip et al., 2018). The current study identifies the mediating role of the self-compassion mechanism that can be applied to organizational research and advances the way attachment impacts workplace outcomes. Specifically, the current findings indicate that attachment styles indirectly affect important organizational outcome behaviors at the workplace, shedding some light on their dynamics. Taken together, the present study offers the construct of self-compassion as a promising mechanism in understanding attachment at the workplace, thus, providing supportive evidence to Neff and Knox’s (2017) conclusion that self-compassion provides the psychological and emotional resilience to cope more successfully with life challenges, including those presented at the workplace. However, the cross-sectional nature of this study cannot be used to determine causal relationships. To determine causal relationships of the investigated variables, future studies might focus on the longitudinal effects of attachment, self-compassion, and different workplace outcomes.

## Author Contributions

AR planned the study, collected and analyzed the data, and wrote the manuscript.

## Conflict of Interest Statement

The author declares that the research was conducted in the absence of any commercial or financial relationships that could be construed as a potential conflict of interest.
